# Association of MCP-1 and CCR2 polymorphisms with the risk of late acute rejection after renal transplantation in Korean patients

**DOI:** 10.1111/j.1744-313X.2007.00725.x

**Published:** 2008-02

**Authors:** S W Kang, S J Park, Y W Kim, Y H Kim, H S Sohn, Y C Yoon, H Joo, K H Jeong, S H Lee, T W Lee, C G Ihm

**Affiliations:** * Department of Nephrology, College of Medicine, Inje University Busan, South Korea; † Department of Preventive Medicine, College of Medicine, Inje University Busan, South Korea; ‡ Department of Chest Surgery, College of Medicine, Inje University Busan, South Korea; § Department of Physiology, College of Medicine, Inje University Busan, South Korea; ¶ Department of Nephrology, College of Medicine, Kyung-Hee University Seoul, South Korea.

## Abstract

Among the factors modulating transplant rejection, chemokines and their respective receptors deserve special attention. Increased expression of monocyte chemoattractant protein-1 (MCP-1) and its corresponding receptor (chemokine receptor-2, CCR2) has been implicated in renal transplant rejection. To determine the impact of the MCP-1-2518G and CCR2-64I genotypes on renal allograft function, 167 Korean patients who underwent transplantation over a 25-year period were evaluated. Genomic DNA was genotyped using polymerase chain reaction followed by restriction fragment length polymorphism analysis. Fifty-five (32.9%) patients were homozygous for the MCP-1-2518G polymorphism. Nine (5.4%) patients were homozygous for the CCR2-64I polymorphism. None of the investigated polymorphism showed a significant shift in long-term allograft survival. However, a significant increase was noted for the risk of late acute rejection in recipients who were homozygous for the MCP-1-2518G polymorphism (OR, 2.600; 95% CI, 1.125–6.012; *P* = 0.022). There was also an association between the MCP-1-2518G/G genotype and the number of late acute rejection episodes (*P* = 0.024). Although there was no difference in the incidence of rejection among recipients stratified by the CCR2-V64I genotype, recipients with the CCR2-V64I GG genotype in combination with the MCP-1-2518G/G genotype had a significantly higher risk of acute or late acute rejection among the receptor-ligand combinations (**P* =* 0.006, *P* = 0.008, respectively). The MCP-1 variant may be a marker for risk of late acute rejection in Korean patients.

## Introduction

Despite advances in immunosuppression and the overall medical care of renal transplant recipients, which have led to an improvement in allograft survival, chronic renal allograft rejection continues to be a major impediment to successful organ transplantation (Milford, 1994). Acute rejection is the single most important risk factor for developing chronic renal allograft rejection (Kamoun, 2001; Ramanathan *et al*., 2001). Donor-recipient HLA mismatches, recipient race, donor age and delayed graft function have been shown to be associated with rejection episodes (Chertow *et al*., 1996). Genetic polymorphisms, other than those at the HLA locus, are attractive factors that might explain some of the clinical heterogeneity with regard to outcome of organ transplantation. Genetic variation may influence the function or expression of key immunoregulatory molecules that mediate transplant rejection.

During acute allograft rejection, monocytes and T-effector cells are directed into the transplant and produce a characteristic tubular or vascular infiltrate (Lalor & Adams, 2001). The complex process of extravasation and influx of leukocyte subsets into the site of tissue injury appears to be mediated, to a significant extent, by the expression of specific chemokines and chemokine receptors (Nelson & Krensky, 2001). Specifically, the expression of the CC-chemokine MCP-1 together with the corresponding chemokine receptor CCR2 can be detected in mononuclear cells infiltrating the kidney graft (Nadeau *et al*., 1995; Pavlakis *et al*., 1996; Grandaliano *et al*., 1997; Nagano *et al*., 1997; Robertson *et al*., 2000; Segerer *et al*., 2001).

Rovin *et al*. (1999) first reported that the MCP-1-2518A/G polymorphism demonstrates ethnic heterogeneity in the general population. The G allele frequency is increased in Asian and Mexican populations compared to European American and African American populations. Kim *et al*. (2002) reported that the A allele, at –2518 of the MCP-1 gene, was associated with the up-regulation of MCP-1, which might result in a more severe proteinuria in Korean patients with lupus nephritis. Krüger *et al*. (2002) reported that the G allele at MCP-1-2518 increased MCP-1 production in immunosuppressed transplant recipients and was a risk factor for kidney allograft failure in patients who underwent kidney transplantation at two centres, one in Germany and one in the United States. These discrepancies may have been caused by differences in the diseases or by ethnic heterogeneity. Although there is to date no evidence that the CCR2-64I polymorphism alters CCR2 expression or function of leukocytes, Abdi *et al*. (2002) reported that significant reductions in risk of acute renal transplant rejection were found in recipients who had the CCR2-64I allele and underwent kidney transplantation in the United States. Therefore, in this study, we investigated whether MCP-1-2518 and CCR2-64I variants are associated with altered kidney allograft outcome in Korean patients.

## Materials and methods

### Patient demographics

A total of 167 first renal allograft recipients transplanted at two centres in Korea (Kyung Hee University Medical Center, Seoul and Busan Baik Hospital, Busan) from 1979 to 2005 were analysed. Demographic data for donor and recipient age and gender, HLA-mismatch, serum creatinine of donor, immunosuppressive therapy, presence of rejection episodes and graft survival were extracted from the hospital record. Acute rejection was determined by allograft biopsy in about 35% of renal transplant rejections. The remaining was defined by an increase in creatinine level by 30% from baseline that was not attributable to other causes with subsequent return to baseline after antirejection therapy. Late acute rejection was defined by the development of acute rejection six months post-transplant. Graft survival was defined as recipient survival with a functioning renal transplant. The Institutional Review Board approved the study, and written informed consent was obtained at the time of enrolment.

### Determination of genotypes

Blood samples for genomic DNA were collected before 2004. Polymerase chain reaction and restriction fragment length polymorphism (PCR-RFLP) study was performed to detect genotypes from the DNA sequence variants of the MCP-1-2518 and CCR2-64I polymorphisms. Genomic DNA was prepared from heparinized venous blood using a Core-one™ blood genomic DNA isolation kit (Corebiosystem, Seoul, Korea). PCR-RFLP was performed as described by Rovin *et al*. (1999) for MCP-1 genotyping. The forward primer was 5′-CCGAGATGTTCCCAGCACAG-3′, and the reverse primer was 5′-TGCTTTGCTTGTGCCTCTT-3′. A total of 20 µL of the reaction mixture contained 200 ng of genomic DNA, 10 pmol of each primer, 200 µm of each dNTP and buffers recommended by the manufacturer. A 930-bp segment of the MCP-5′-flanking region between nucleotide –1817 and –2746, relative to the major transcriptional start site defined by Ueda *et al*. (1994), was amplified by GeneAmp PCR system 9600 (Applied Biosystems, Foster City, CA, USA) under the following conditions: 35 cycles of 30 s at 94 ºC, 30 s at 64 ºC and 30 s at 72 ºC. The extension time of the last cycle was set at 7 min. PCR products were digested by the corresponding restriction enzyme Pvu II (Takara Shuzo Co, Kyoto, Japan) for 16 h at 37 ºC. Digestive products were separated from 1% agarose gels stained with ethidium bromide. Pvu II digested the 930-bp DNA segment from G/G homozygous individuals into 708- and 222-bp fragments. DNA from A/A homozygous individuals was not cut with Pvu II. DNA from A/G heterozygous individuals showed the expected fragments at 930, 708 and 222 bp.

CCR2-V64I genotyping was performed as originally described by Smith *et al*. (1997) with some minor modifications. PCR was performed as described previously except for the use of an annealing temperature of 57 °C. The forward primer was 5′-ATTTCCCCAGTACATCCACAAC-3′, and the reverse primer was 5′-CCCACAATGGGAGAGTAATAAG-3′. PCR cycles were as follows: 95 °C for 3 min followed by 35 cycles each of 95 °C for 30 s, 57 °C for 30 s and 72 °C for 30 s. Amplification resulted in a 317-bp product, 10 µL of which was then digested in a 20-µL reaction for 16 h with 1 unit of Fok I (New England BioLabs, Beverly, MA, USA) per the manufacturer's recommendations. An A at nucleotide position 190 encodes isoleucine at amino acid position 64 and yields restriction fragments of 197 and 120 bp after Fok I digestion. By contrast, the 317-bp amplicon remains uncut if a G encoding a valine is present.

### MCP-1 production

MCP-1 released from unstimulated and stimulated peripheral blood mononuclear cells (PBMC) was determined by enzyme-linked immunosorbent assay (ELISA) as described previously (Rovin *et al*., 1999). Briefly, PBMC from kidney transplant recipients, a total of 22 patients (A/A 6, A/G 9, G/G 7), were isolated by density gradient centrifugation. PBMCs were cultured for 24 h with and without addition of 10 ng mL^−1^ IL-1 (R & D Systems, Minneapolis, MN, USA). MCP-1 concentrations were assayed in cell-free supernatants by specific ELISA according to the manufacturer's instructions (Quantikine; R & D Systems).

### Statistical analyses

Patient genotypes were used as categorical independent variables for analyzing continuous outcome variables (graft survival), the binary variable (presence or absence of a rejection episode) and the ordinal variable (number of acute rejection episodes). Nominal logistic fit was used to assess the association of genotypes with the incidence of acute rejection. anova was used when the effect of two independent variables (factors) on a dependent numerical variable was examined. Graft survival was analysed with Kaplan-Meier estimates. Groups were compared with the log rank test. Multivariate analysis for the respective risk factors of acute rejection or graft survival was performed with logistic regression or Cox regression. Fisher's exact test was used to test for differences in immunosuppressive therapy between the groups. A *P* < 0.05 was considered significant. The statistical software package (sas 9.1 for Windows) was used for the calculations.

## Results

### Patient demographic and clinical data

The genotype frequencies of MCP-1-2518A/G and CCR2-V64I polymorphisms were as follows. For the MCP-1-2518 genotypes among the 167 kidney transplanted patients, 21 had the A/A genotype (12%), 91 the A/G type (55%) and 55 the G/G type (33%). We could get only 166 PCR products for CCR2 and the remaining one was not amplified; then, we genotyped only 166 patients for CCR2. For the CCR2-V64I genotypes, among the 166 kidney transplanted patients, 9 had the A/A genotype (5%), 84 the G/A type (51%) and 73 the G/G (44%). The overall incidences of acute rejection and late acute rejection were 31.7% and 16.2%, respectively. Total number of acute rejection episodes was 85. Biopsy was carried out in 30 of 85 episodes, and 26 cases (87%) among the biopsy procedures were confirmed as acute rejection. Individuals with or without the MCP-1-2518G/G genotype did not differ with respect to other risk factors for rejection frequency and allograft failure such as donor age, ethnic background, living donor, HLA-mismatch, immunosuppressive therapy, recipient age and gender and the serum creatinine level of the donor. However, individuals with the MCP-1-2518G/G genotype had a higher occurrence of late acute rejection than individuals with other genotypes (Table 1).

**Table 1:** Demographic and clinical data for 167 kidney transplanted patients with or without the MCP-1-2518G/G genotype

	MCP-1-2518		
	A/A or A/G	G/G	P
Number (M: F)	112 (72 : 40)	55 (41 : 14)	0.129
Recipient age (years)	37.0 ± 10.6	33.8 ± 11.3	0.079
Donor age (years)	39.9 ± 14.4	40.1 ± 13.2	0.936
Cadaveric donors	17 (15.3%)	8 (14.5%)	0.925
HLA-mismatch (total number)	2.8 ± 1.1	2.8 ± 1.5	0.762
Calcineurin inhibitors	92%	98%	0.209
Anti IL-2 receptor antibody	24%	20%	0.690
Donor serum creatinine (mg dL^−1^)	1.0 ± 0.5	0.9 ± 0.2	0.350
Acute rejection (total)	26.8%	41.8%	0.054
Late acute rejection (after 6 months)	11.6%	25.5%	0.022
Graft survival time (months)	89.1 ± 67.9	85.6 ± 67.3	0.762

### Univariate analysis and multivariate analysis of risk factors for late acute rejection

The univariate analysis of variables related to the occurrence of late acute rejection is shown in Table 2a. The variables related to the occurrence of late acute rejection were recipient age, donor age, number of HLA-mismatch, donor serum creatinine level and MCP-1-2518 GG genotype (respective *P* values are shown in Table 2a). Total numbers of patients with and without late acute rejection episodes were 27 and 140, respectively.

**Table 2:** Univariate analysis(a) and multivariate logistic regression analysis(b) of variables predicting risk for late acute rejection

(a)					

Variables	Rejection *N* (%)	No rejection *N* (%)	OR*	95%C.I.*	P
Recipient gender (M:F)	20 : 7	93 : 47			0.437
Recipient age (years)	33.0 ± 12.4	36.5 ± 10.6			< 0.0001
Donor age (years)	42.0 ± 12.3	39.6 ± 14.4			< 0.0001
Number of HLA-mismatch	2.96 ± 1.02	2.80 ± 1.37			< 0.0001
Donor serum creatinine (mg dL^−1^)	0.96 ± 0.15	0.99 ± 0.48			< 0.001
Cadaveric donors	4 (14.8%)	21 (15.1%)	0.98	0.32–3.26	0.969
Calcineurin inhibitors	25 (92.6%)	124 (89.2%)	1.51	0.33–7.03	0.598
Anti IL-2 receptor antibody	4 (14.8%)	34 (24.3%)	0.54	0.18–1.68	0.288
GG at MCP-1-2518	14 (52.9%)	41 (29.3%)	2.60	1.13–6.01	0.025

(b)			

Variables	OR^a^	95% C.I.^b^	P
Recipient age (years)	0.99	0.95–1.03	0.565
Donor age (years)	1.02	0.98–1.05	0.382
Number of HLA-mismatch	1.06	0.77–1.47	0.714
Donor serum creatinine (mg dL^−1^)	0.90	0.27–3.02	0.858
GG at MCP-1-2518	2.63	1.05–6.58	0.043

* OR; odds ratio

* C.I.; confidence interval.

a OR; odds ratio

b C.I.*; confidence interval.

To verify the results of the univariate analysis, a multivariate logistic regression analysis using the variables found significant in the univariate analysis was performed. The multivariate logistic regression analysis revealed that only the presence of the G/G genotype was associated with the occurrence of late acute rejection (Table 2b).

### Association of the polymorphism with acute or late acute rejection

The relationship of the patients’ MCP-1-2518 or CCR2-V64I genotypes with the incidence of renal allograft rejection was analysed. The percentage of recipients who had a late acute rejection episode was more than twofold higher in individuals with a MCP-1-2518G/G genotype vs. A/A or A/G genotypes (25.5% for GG vs. 11.6% for AA or AG; odds ratio [OR], 2.60; 95% CI, 1.13–6.01; *P* = 0.022). There was no difference in the incidence of rejection among recipients stratified by the presence or absence of the CCR2-64I allele (*P* = 0.334). There was also a correlation between the MCP-1-2518 GG genotype and the number of late acute rejection episodes (*P* = 0.024) (Table 3).

**Table 3:** MCP-1 genotype and number of acute or late acute rejection episodes

		Number of rejections			
			
Polymorphism (MCP-1-2518)	Genotype	0	1	> 1	P
AR^a^	G/G	32	14	9	0.139
	A/A & A/G	82	17	13	
LAR^b^	G/G	41	10	4	0.024
	A/A & A/G	99	12	1	

a AR; acute rejection

b LAR; late acute rejection.

To explore whether consequences of genetically determined alterations of receptor–ligand interactions in the chemokine system would better identify high-risk recipients, further analysis of combinations of these receptor–ligand gene polymorphisms was performed (Table 4a and b). Recipients with the CCR2-V64I GG genotype in combination with the MCP-1-2518G/G genotype had a significantly higher risk for acute or late acute rejection among the combinations (OR = 4.62, *P* = 0.006 or OR = 7.06, *P* = 0.008). In Table 4, compared with MCP-1-2518 AA or AG and CCR2-V64I GG, all other combinations seem associated with acute rejection, if the arbitrarily conventional significance of *P* < 0.05 is not rigidly followed.

**Table 4:** Effect of combinations of chemokine receptor-ligand gene polymorphism on acute or late acute rejection episodes

(a)					

Polymorphism					
				
CCR2-V64I	MCP-1-2518	AR^a^ (%)	OR^b^	95% C.I.^c^	P
G/G	AA/AG	8/48 (16.7)	1		
G/G	G/G	12/25 (48.0)	4.62	1.55–13.75	0.006
GA/AA	AA/AG	21/63 (33.3)	2.50	0.99–6.29	0.052
GA/AA	G/G	11/30 (36.7)	2.89	1.00–8.37	0.050

(b)					

Polymorphism					
				
CCR2-V64I	MCP-1-2518	LAR^a^ (%)	OR^b^	95% C.I.^c^	P
G/G	AA/AG	3/48 (6.3)	1		
G/G	G/G	8/25 (32.0)	7.06	1.67–29.78	0.008
GA/AA	AA/AG	9/63 (14.3)	2.50	0.64–9.80	0.188
GA/AA	G/G	6/30 (20.0)	3.75	0.86–16.33	0.078

a AR; acute rejection

b OR; odds ratio

c C.I.; confidence interval.

a LAR; late acute rejection

b OR; odds ratio

c C.I.; confidence interval.

### Statistical analysis of risk factors for graft failure

MCP-1 and CCR2 genotypes were not significant risk factors for allograft failure in the univariate analysis, nor the combinations of MCP-1 and CCR2 polymorphism. According to multivariate analysis of risk factors for graft failure, factors such as donor age, HLA-DR mismatch, donor serum creatinine, and MCP-1-2518 GG genotype did not give risks for graft failure. However, younger recipient group and the occurrence of late acute rejection gave significant risks for allograft failure because respective odds ratio and *P* value of recipient age or the occurrence of late acute rejection were as follows; odds ratio for recipient age, 0.911; *P* = 0.001 or odds ratio for the occurrence of late acute rejection, 6.48; *P* = 0.002.

### Effect of genotypes on graft survival

A total of 30 grafts (18%) lost function finally; the mean graft survival was 88 months. In accordance with a previous study (Krüger *et al*., 2002), the G/G genotype was compared with the combined A/A and A/G genotypes. The graft survival using the Kaplan–Meier estimation showed no significant difference between the two genetic groups. In addition, the 64I polymorphism of CCR2 was not associated with kidney graft survival.

Multivariate graft survival analysis of patients with acute rejection episodes (*n* = 53) was performed. Thirty-six of 53 patients had functioning renal transplant. The results showed that the MCP-1-2518G/G recipients with rejection episodes had poorer graft survival compared with the A/A or A/G recipients with rejection episodes after multivariate correction for the risk factors listed in Table 5. The Cox proportional hazard analysis showed that the G/G genotype was one of the independent risk factors for graft failure in patients with acute rejection episodes, in addition to other risk factors such as donor age, donor serum creatinine level at pre-operation and the number of HLA-DR mismatches. The G/G genotype was associated with a 2.27-fold risk for graft failure in patients with acute rejection episodes (95% CI; 1.19–4.35; *P* = 0.014; Table 5). There was no difference in graft survival between the G/G and A positive recipients without rejection episodes (data not shown).

**Table 5:** Cox proportional hazard model to test the significance of clinical covariates and genotypes of MCP-1 polymorphism as predictors of graft failure with acute rejection episodes

Variable	OR^a^	95% C.I.^b^	*P*-value
Donor age (years)	1.02	1.000–1.045	0.056
Recipient age (years)	1.01	0.983–1.039	0.449
HLA-DR mismatch	2.46	1.165–5.206	0.019
Donor serum creatinine (mg dL^−1^)	2.25	1.022–4.493	0.045
GG genotype at MCP-1-2518	2.27	1.189–4.346	0.014

a OR; odds ratio

b C.I.; confidence interval.

### MCP-1 production

**Figure 1:**
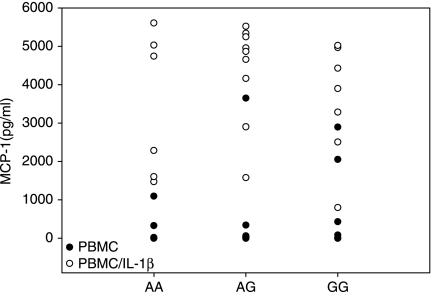
MCP-1 levels in the culture supernatant of peripheral blood mononuclear cells. Mononuclear cells were isolated from the peripheral blood of patients, and cultured with 10 ng mL^−1^ IL-1β. The level of MCP-1 protein in the culture supernatant was determined by sandwich ELISA.

The effect of the –2518G-allele on MCP-1 production after stimulation with the pro-inflammatory cytokine IL-1β was examined. No significant differences were noted between allelic types after IL-1β stimulation (Fig. 1). At the time of blood collection, the two groups studied for MCP-1 production did not differ with regard to immunosuppressive therapy (*P* = 0.74 by Fisher's exact test).

## Discussion

Multiple associations have been reported with gene polymorphisms of HLA, cytokine, and costimulatory molecules and clinical outcomes of kidney transplantation (Akalin & Murphy, 2001). Both the CCR2 and the CCR2 ligand, MCP-1, have been shown to be markedly up-regulated in renal transplant rejection (Grandaliano *et al*., 1997; Robertson *et al*., 2000; Segerer *et al*., 2001). In addition, MCP-1 is an important chemoattractant for monocytes; it is possible that this polymorphism may affect the migration of monocytes into the rejecting graft. Few studies, however, have explored the consequences of genetically determined alterations of receptor–ligand interactions in the chemokine system.

The results of this study demonstrated an association between MCP-1 polymorphisms and late acute renal allograft rejection. The proportion of patients with late acute rejection as well as the number of rejection episodes per patient was significantly higher in patients with the MCP-1-2518G/G allele. In contrast to previous results (Krüger *et al*., 2002) the present study showed no significant association of allograft failure with the G/G allele of the MCP-1-2518 polymorphism. Our functional data also showed that PBMCs isolated from kidney transplant recipients who carried this allele had a similar production of MCP-1 to other alleles. However, the functional data may have been affected by immunosuppressive therapy. The main purpose in this investigation was to determine whether there was a difference in graft survival after transplantation in two genetically different groups among Koreans with potentially different MCP-1 production. Significant differences were identified only in patients with rejection episodes, whereas in patients without rejection episodes no differences were noted. These results suggest a possible modifying affect of the MCP-1 polymorphism on the course of graft rejection, or possibly sensitivity to treatment affecting graft survival. Because the rejection frequency, but not the graft survival, was observed to be different in this study, we suggest that this association may be caused by a modifying affect of the MCP-1 gene on the initiation of the rejection reaction but not on the severity.

This graft failure appears to be associated with the rejection episode and not solely to the presence of the MCP-1-2518 GG allele; this is supported by the finding that there was no difference in graft survival between the groups of recipients without rejection episodes, and that there were very few grafts lost among recipients without acute rejection episodes. These findings do not support the G/G allele as an independent risk factor. Although not clearly apparent from the clinical parameters used to classify rejection episodes, the G/G recipients had a higher risk of developing rejection that led to graft loss. However, the A/A and A/G recipients with rejection episodes were more likely to develop benign rejection episodes; these episodes did not lead to loss of the graft during follow-up. The A/A and A/G recipients were more like the recipients without rejection episodes. These results provide an explanation with the genetic background added to earlier observations that showed that severity, recurrence and steroid resistance were associated with rejection episodes and decreased graft survival (Cecka & Terasaki, 1989; Gulanikar *et al*., 1992; Vanrenterghem, 1995).

Although there was no difference in the incidence of rejection among recipients stratified by the CCR2-V64I genotype in this study, recipients with the CCR2-V64I GG genotype in combination with the MCP-1-2518G/G genotype had a significantly higher risk of acute or late acute rejection among the receptor–ligand combinations (**P* =* 0.006, *P* = 0.008, respectively). Therefore, we hypothesize that the CCR2 axis may promote the migration of monocytes into the transplanted kidney; these pairs of chemokines and their receptors may act sequentially or simultaneously to facilitate migration and fine positioning of cells expressing the chemokine receptor. Furthermore, this association suggests that interactions between the chemokine receptor polymorphism with the ligand polymorphism (gene × gene) may control disease susceptibility and progression.

Because of the small size of this cohort and its retrospective nature, this study should be considered preliminary. Future studies will be needed to confirm these chemokine receptor–ligand polymorphism associations with transplant rejection incidence as well as to further clarify their mechanism of action. Precise delineation of how the chemokine system functions in renal transplantation rejection may point to new therapeutic targets and prognostic markers. There was no consideration of the donor genotype in this study, although the interaction between cells in the graft and recipient leukocytes would presumably have an influence on rejection. Hoffmann *et al*. (2004) demonstrated that donor cytokine polymorphisms can affect the likelihood of both acute rejection and, perhaps more strongly, the development of chronic allograft nephropathy. Future study will be needed to find the affect of donor's MCP-1 and CCR2 polymorphisms on transplant outcome.

It is likely that there are different regulatory features in different ethnic groups. The higher incidence of the G allele compared to the A allele in Koreans, and a similar level of MCP-1 production from both alleles, are in contrast with previous findings in European Americans and Afro Americans (Rovin *et al*., 1999; Krüger *et al*., 2002). Such a discrepancy may be caused by distinct factors affecting the levels of MCP-1 production in populations with different genetic backgrounds, possibly as a result of additional polymorphisms at other genetic loci affecting the cellular functions of MCP-1. Thus, studies involving larger and ethnically diverse samples are needed to better understand functional polymorphisms and to determine whether they are linked with other genes or interact with other genes, or are susceptibility markers.

Recipients of renal transplants with the MCP-1-2518G/G genotype are at increased risk for late acute rejection. This variant of MCP-1 may provide a novel marker for late acute rejection in Korean patients.

## Abbreviations

CCR2: chemokine receptor-2
ELISA: enzyme-linked immunosorbent assay
MCP-1: monocyte chemoattractant protein-1
PCR-RFLP: polymerase chain reaction and restriction fragment length polymorphism
PBMCs: peripheral blood mononuclear cells
